# Bioanalytical and Mass Spectrometric Methods for Aldehyde Profiling in Biological Fluids

**DOI:** 10.3390/toxics7020032

**Published:** 2019-06-04

**Authors:** Romel P. Dator, Morwena J. Solivio, Peter W. Villalta, Silvia Balbo

**Affiliations:** Masonic Cancer Center, University of Minnesota, 2231 6th Street SE, Minneapolis, MN 55455, USA; rpdator@umn.edu (R.P.D.); msolivio@umn.edu (M.J.S.)

**Keywords:** aldehydes, genotoxicity, cancer, diseases, oxidative stress, exposure biomarkers, high-resolution mass spectrometry, data-dependent profiling, derivatization, biological fluids, isotope labeling

## Abstract

Human exposure to aldehydes is implicated in multiple diseases including diabetes, cardiovascular diseases, neurodegenerative disorders (i.e., Alzheimer’s and Parkinson’s Diseases), and cancer. Because these compounds are strong electrophiles, they can react with nucleophilic sites in DNA and proteins to form reversible and irreversible modifications. These modifications, if not eliminated or repaired, can lead to alteration in cellular homeostasis, cell death and ultimately contribute to disease pathogenesis. This review provides an overview of the current knowledge of the methods and applications of aldehyde exposure measurements, with a particular focus on bioanalytical and mass spectrometric techniques, including recent advances in mass spectrometry (MS)-based profiling methods for identifying potential biomarkers of aldehyde exposure. We discuss the various derivatization reagents used to capture small polar aldehydes and methods to quantify these compounds in biological matrices. In addition, we present emerging mass spectrometry-based methods, which use high-resolution accurate mass (HR/AM) analysis for characterizing carbonyl compounds and their potential applications in molecular epidemiology studies. With the availability of diverse bioanalytical methods presented here including simple and rapid techniques allowing remote monitoring of aldehydes, real-time imaging of aldehydic load in cells, advances in MS instrumentation, high performance chromatographic separation, and improved bioinformatics tools, the data acquired enable increased sensitivity for identifying specific aldehydes and new biomarkers of aldehyde exposure. Finally, the combination of these techniques with exciting new methods for single cell analysis provides the potential for detection and profiling of aldehydes at a cellular level, opening up the opportunity to minutely dissect their roles and biological consequences in cellular metabolism and diseases pathogenesis.

## 1. Introduction

### Sources of Human Exposure to Aldehydes

**Figure 1:**
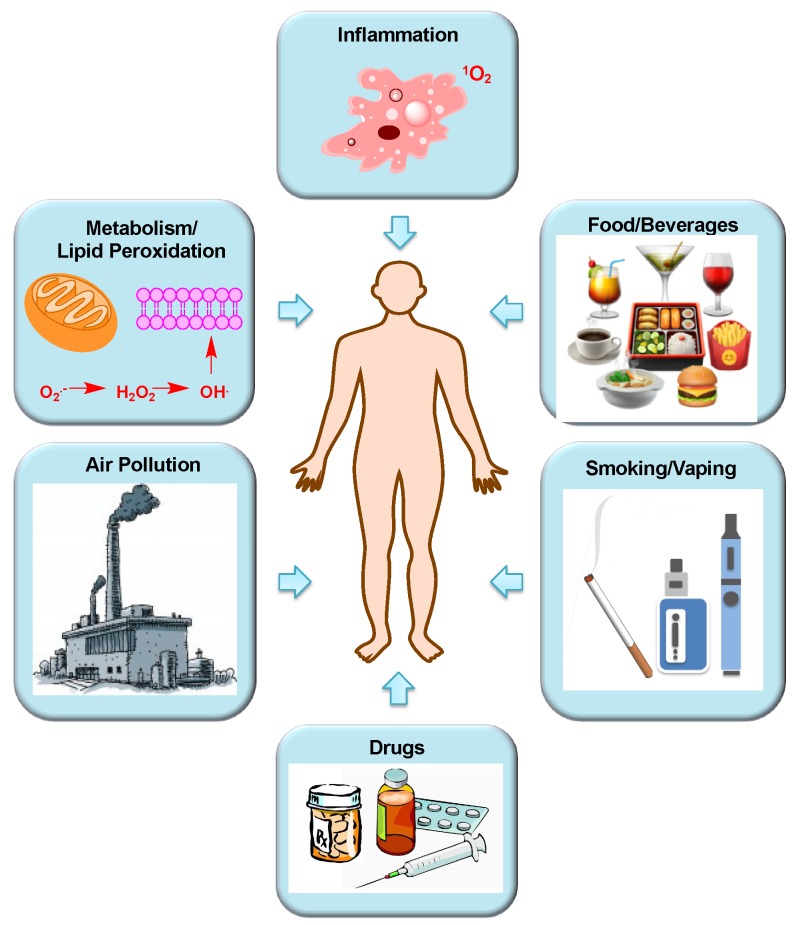
Exogenous and endogenous sources of human exposure to aldehydes.

Aldehydes are characterized by the presence of a –HC = O reactive site and often exist in combination with other functional groups. They are ubiquitous in the environment, originating from man-made sources, as well as through natural processes (Figure 1). The hydroxyl radical mediated-photochemical oxidation of hydrocarbons generates aldehydes in the atmosphere [1,2,3]. For instance, formaldehyde is produced from the oxidation of methane and naturally occurring compounds, such as terpenoids and isoprenoids from tree foliage [2]. In industrialized areas, the majority of aldehydes are produced from motor vehicle exhaust (internal diesel engine combustion), which either directly yields aldehydes or generates hydrocarbons, which are eventually converted to aldehydes by photochemical oxidation reactions [1,4,5,6,7,8]. Formaldehyde, acetaldehyde, and acrolein are significant contributors to the overall summed risk of mobile sources of air toxicants according to the United States Environmental Protection Agency (U.S. EPA) [1]. Other sources of aldehydes include agricultural and forest fires, incinerators, and coal-based power plants [9,10,11,12,13]. Additionally, humans are exposed to aldehydes in residential and occupational settings where aldehydes are present in confined spaces [14] due to the release of fumes from indoor furniture, carpets, fabrics, household cleaning agents, cosmetic products, and paints [12,15,16,17,18]. Aldehydes are also widely used as fumigants and for biological specimen preservation [1]. Another major source of aldehyde exposure comes from cigarette smoke. Mainstream tobacco smoke (MTS) is composed of significant amounts of acetaldehyde as the major component, followed by acrolein, formaldehyde, and crotonaldehyde [19,20,21,22,23,24,25,26]. Similarly, popular devices such as e-cigarettes, which are advocated as safer alternatives to tobacco, have been found to generate high concentrations of aldehydes [27,28,29,30,31,32,33,34,35,36,37]. Aldehydes are also present in food and beverages (as flavorings), and in alcoholic drinks either as congeners or, in the case of acetaldehyde, as the oxidative by-product of ethanol [38,39,40]. Biotransformation is another source of aldehyde exposure. This includes metabolism of a sizeable number of environmental agents, such as drugs, tobacco smoke, alcohol, and other forms of xenobiotics [41,42,43]. Of note, exposure also comes from the metabolism of a number of widely used anticancer drugs such as cyclophosphamide, ifosfamide, and misonidazole as well as other drugs used for the treatment of diseases such as epilepsy and HIV-1 infection [1]. The production of aldehydes is proposed to be an important contributor to the toxicity and undesirable side effects of treatment with these drugs.

Finally, normal cellular metabolic pathways such as lipid peroxidation, Alk-B type repair, histone demethylation, carbohydrate or ascorbate autoxidation, carbohydrate metabolism, and amine oxidase-, cytochrome P-450-, and myeloperoxidase-catalyzed metabolic pathways produce aldehydes endogenously [1,44,45]. The metabolism of molecules such as amino acids, vitamins, and steroids, to name a few, also generates aldehydes [46]. Aldehydes are generally formed during conditions of high oxidative stress. Oxidants are generated as a result of normal intracellular metabolism in the mitochondria, peroxisomes, and a number of cytosolic enzyme systems [47]. These metabolic free radicals and oxidants are referred to as reactive oxygen species (ROS). A balance between ROS production and removal by the antioxidant defense systems is essential to maintaining redox homeostasis. A disturbance in the balance favoring pro-oxidative conditions results to oxidative stress. An elevated level of ROS and the resulting oxidative stress leads to biological damage and is implicated in aging and pathologies of various conditions including cancer, cardiovascular, inflammatory, and neurodegenerative diseases [47,48]. The generation of aldehydes is one important consequence of sustained oxidative stress, which can result to the auto-oxidation of lipids (damaging cell membranes) and fatty acids within cells. Lipid peroxidation occurs when a variety of ROS and/or reactive nitrogen species (RNS) oxidize lipids containing carbon-carbon double bonds, especially polyunsaturated fatty acids, resulting in free radical chain reactions and subsequent formation of by-products such as lipid radicals, hydrocarbons, and aldehydes [49]. The correlation between elevated ROS and aldehyde production has been extensively studied and is known to contribute to a multitude of disease pathologies by altering proteomic, genomic, cell signaling, and metabolic processes [50,51]. Indeed, 4-hydroxy-2-nonenal (4-HNE) and malondialdehyde (MDA) are both used as markers of the magnitude of oxidative stress and lipid peroxidation [52]. Dietary consumption of polyunsaturated fatty acids and the subsequent oxidation of these molecules can also result to the formation of aldehydes. The carbohydrate or ascorbate autoxidation pathways generate endogenous glyoxal, which is a major lipid and DNA oxidative degradation product [1]. Likewise, methylglyoxal is produced through the enzymatic reactions of triose phosphate intermediates such as glyceraldehyde-3-phosphate and dihydroxyacetone phosphate during glycolysis or from the metabolism of ketone bodies or threonine [53]. The serum amine oxidase (SAO) and polyamine oxidase (PAO) also generate endogenous aldehydes by catalyzing the deamination of biogenic amines [1]. In summary, the dysregulation of metabolic processes and oxidative stress result in lipid peroxidation, carbohydrate auto-oxidation, protein oxidation, as well as polyamine catabolism, all result in aldehyde formation.

## 2. Biological Consequences of Aldehyde Exposure on Genome Integrity, Carcinogenesis, and Other Diseases

**Figure 2:**
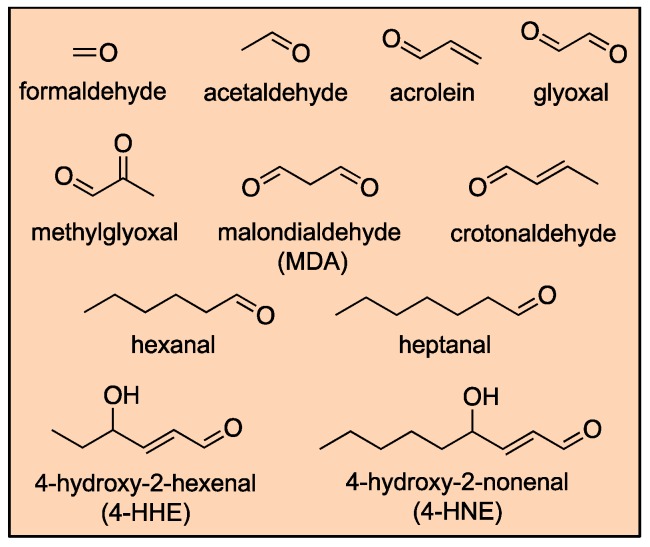
Structures of common aldehydes associated with various human diseases.

Low molecular weight aldehydes such as formaldehyde, acetaldehyde, and acrolein are generally toxic compounds. The majority of the most abundant aldehydes are irritants at high doses, and, due to their volatility, induce acute inhalation toxicity. Additionally, aldehydes are believed to play major roles in various debilitating diseases such as cancer and neurodegeneration. Aldehydes are highly reactive, electrophilic compounds, which can exert their detrimental role through interactions with various biomolecules such as phospholipids, peptides, regulatory proteins, enzymes, and DNA forming covalent modifications, affecting their normal functions and leading to mutations and chromosomal aberrations. These mediated effects vary from physiological and homeostatic, to cytotoxic, mutagenic, and carcinogenic [54,55]. Figure 2 shows the structures of common aldehydes implicated in the pathogenesis of multiple human diseases.

Formaldehyde and acetaldehyde, from alcohol consumption, have been classified as Group 1 human carcinogens by the International Agency for Research on Cancer (IARC) [56,57,58,59]. Both compounds are believed to exert their carcinogenic effects by reacting with DNA, forming covalent modifications known as DNA adducts [60,61,62,63,64,65,66]. These adducts if not repaired or eliminated may translate into mutations and ultimately into dysregulation of normal cellular growth. Aldehyde toxicity is also implicated in aging, and age-related diseases such as cardiovascular and neurological disorders [67,68,69,70]. Unlike free radicals with shorter half-lives ranging from nanoseconds to milliseconds, reactive carbonyl compounds (RCCs) including aldehydes are more stable with half-lives ranging from minutes to hours. Because of this relative stability, aldehydes are long-lived and can therefore diffuse from the point of origin and intracellularly and extracellularly attack targets, which are distant from the radical events [71,72].

Mounting evidence indicates that endogenous aldehydes, such as MDA, 4-HNE, 3-aminopropanal (3-AP), acrolein, formaldehyde, and methylglyoxal, are mediators of neurodegeneration [73] and aldehydes formed during lipid peroxidation (advanced lipoxidation end-products, ALEs) and sugar glycoxidation (advanced glycoxidation end-products, AGEs) accumulate in several oxidative stress and aging disorders [74]. Aldehydes foster oligomerization of proteins and peptides found in neuritic plaques, which is a characteristic of Alzheimer’s disease (AD) [75,76,77]. Physiological concentrations of these aldehydes range from nM to several hundred μM [78,79]. Methylglyoxal concentration in human blood is estimated to be in the 100–120 nM range, while its cellular concentration is about 1–5 μM and 0.1–1 μM for glyoxal [80,81,82]. MDA concentration in serum is 0.93 ± 0.39 μM [83] and 4-HNE concentration in cells is less than 1 μM [52]. Likewise, the levels of acrolein formed by metabolism are hard to quantify and may reach very high levels in certain microenvironments [84]. Increased levels of these aldehydes in brain and cerebrospinal fluid (CSF) were reported for various neurodegenerative disorders [85]. The levels of 4-HNE are found to increase in the brain regions of deceased AD patients compared to age-matched controls [86], and are elevated in CSF of AD patients compared to healthy controls [87]. Likewise, acrolein is found to be elevated in the amygdala and hippocampus/parahippocampal gyrus in brains of AD patients compared to controls [88]. Protein carbonylation has been associated with the progression of several neurodegenerative disorders including AD, Parkinson’s disease (PD), multiple sclerosis (MS) and amyotrophic lateral sclerosis (ALS).

Methylglyoxal is found at significantly higher levels in diabetic patients compared to healthy controls [89], while 4-HNE, is known to form adducts with mitochondrial proteins, (specifically through interactions with cysteine, histidine, and lysine residues), lipids, and DNA resulting to mitochondrial malfunction. The mitochondrial electron transport chain is the most important source of endogenous ROS, converting 1–2% of the total oxygen consumed into superoxide anions [90,91]. An estimate of 1–8% of 4-HNE produced in cells will form adducts with proteins, with 30% of it occurring in the mitochondria, making it consequential in ROS production [51,92,93]. In some cases, ROS overproduction has been associated with mutations in a mitochondrial gene that encodes a component of the electron transport chain [94]. Increasing damage to mitochondrial DNA inevitably results to compromised mitochondrial function and integrity, leading to a vicious cycle of ROS generation and DNA damage [91]. Oxidative damage to mitochondrial DNA in the heart and the brain has been shown to decrease the lifespan in mammals, and mitochondrial dysfunction has been associated with some neurological disorders including AD, PD, Huntington’s Diseases (HD), and ALS [48,95].

Finally, endogenous aldehydes may also play a role in the free radical theory of aging at the molecular level, which has gained widespread attention and acceptance. In this context, aging is viewed as a process related to an imbalance favoring pro-oxidant over antioxidant molecules (either by ROS elevation or an age-related downregulation of antioxidant molecules and ROS-mitigating enzymes) and consequently an increase in oxidative stress and the level of aldehydes resulting from it [72].

Despite the fact that these molecules fundamentally underlie early events driving the initiation and propagation of various pathologies, their exact role and diagnostic or prognostic value as clinical biomarkers have been underexploited [96]. The complete cellular “aldehydic load” is considered an important parameter for appraisal of these pathologic statuses [97,98]. Developing methods to detect free aldehydes in biological systems is important in understanding the roles and functions of these molecules in cellular processes and disease pathogenesis. The measurement of free aldehydes has the potential to be used to characterize exposure, but also to identify biomarkers for early disease diagnosis, monitor disease progression and response to therapy, and investigate physiological malfunctions such as high oxidative stress.

## 3. Metabolism of Aldehydes

As outlined in the previous section, excessive exposure to aldehydes can result in the disruption of a number of cellular functions, which can ultimately contribute to human diseases. The balance between the activation and detoxification of aldehydes will dictate their toxicity, which is dependent on the aldehyde itself and the presence of aldehyde metabolizing enzymes in cells. Several metabolic pathways and metabolizing enzymes are responsible for the metabolism and detoxification of aldehydes. These enzymes include aldehyde-oxidizing enzymes, aldehyde-reducing enzymes, and glutathione (GSH)-dependent aldehyde metabolizing enzymes, as previously reviewed by O’Brien [1]. For instance, 4-HNE is metabolized by glutathione S-transferase (GST) and aldehyde dehydrogenase 2 (ALDH2), and to a minor extent alcohol dehydrogenase (ADH) in rat hepatocytes [92,99,100,101,102]. Methylglyoxal is likely metabolized by glyoxalase (GLOX) and reduced by aldo-keto reductase (AKR) 1A2 [1]. The inhibition of ALDH2 activity, with the consequent increase in the level of aldehydes by oxidative stress was also observed in humans and diabetic mice during aging and is associated with cardiac dysfunction [103]. Elimination and in vivo metabolism of alkanals and aromatic aldehydes is via dehydrogenase-catalyzed oxidation. Likewise, the main in vivo elimination and metabolism of alkenals such as acrolein is via glutathione conjugation catalyzed by glutathione transferases [1].

In the case of formaldehyde, its metabolism is known to be mediated by alcohol and aldehyde dehydrogenases, ADH5 and ALDH2, respectively. Depletion of GSH levels in hepatocytes and inhibition of these enzymes result in a marked increase in formaldehyde cytotoxicity [104]. Formaldehyde is a potent DNA and protein cross-linking molecule that organisms produce in vast quantities, through one carbon metabolism (1C-metabolism), and in processes such as enzymatic demethylation of histones and nucleic acids [105]. This is supported by the blood formaldehyde concentration, which ranges from 20–100 μM, and 200–400 μM in a healthy human brain, indicating a substantial source of this molecule [106,107,108,109]. A study on mice revealed a two-tier protection mechanism, shielding mice from high levels of endogenous formaldehyde. The first tier involved the enzyme ADH5, which eliminates formaldehyde, while the Fanconi Anemia pathway for cross-link repair reverts DNA damage due to formaldehyde. It was hypothesized that ADH5-dependent formaldehyde oxidation into formate could provide 1C units to enable nucleotide synthesis [110]. Formaldehyde reacts spontaneously with intracellular GSH, present in substantial amounts to form S-hydroxymethylglutathione (HMGSH), which undergoes oxidation by ADH5 and NAD(P)^+^ to generate S-formylglutathione (FGSH), which is subsequently converted by S-formylglutathione hydrolase (FGH) regenerating GSH and yielding formate. The formate formed in this process is eventually used in biosynthetic reactions [111], thus showing that formaldehyde detoxification produces a 1C unit sustaining essential metabolism [55], including the biosynthesis of purines and thymidine, homeostasis of amino acids glycine, serine, and methionine, epigenetic maintenance, and redox defense [112]. This biochemical route of formaldehyde detoxification can therefore provide the cell with utilizable 1C units [111]. Since this genotoxic molecule is generated in large amounts in the human body, a steady-state balance between formaldehyde generation and removal is established due to detoxification by cellular enzymes including alcohol dehydrogenase 1 (ADH1), which reduces cytosolic formaldehyde to methanol, mitochondrial ALDH2, cytosolic alcohol dehydrogenase 3 (ADH3), also known as glutathione-dependent formaldehyde dehydrogenase, as well the previously mentioned ADH5, all responsible for formaldehyde metabolism [113,114,115,116].

Aldehydes are oxidized by the aldehyde dehydrogenase superfamily, of which 16 genes and 3 pseudogenes have been identified in the human genome, including ALDH1A, ALDH2, ALDH1B1, ALDH3A1, and ALDH3A2. ALDH2, for example, is efficient at metabolizing acetaldehyde, a reactive metabolite of ethanol, to acetate and likely plays a major role in reducing the toxicity of aldehydes in humans [117]. Likewise, the aldehyde-reducing enzymes are another superfamily of enzymes responsible for the reduction of aldehydes to alcohol using NADH as a cofactor, and which can be divided into several classes corresponding to the necessary cofactors. The ADH superfamily preferentially uses NADH to reduce aldehydes to alcohols, while using NAD+ to do the reverse reaction but to a lesser extent [1]. This class of enzymes is located in the cytosol and includes ADH1, ADH2, and ADH3. The aldo-keto reductase superfamily uses NADPH solely while others use both NADPH and NADH. This class of enzymes includes AKR1A1, AKR1C, and AKR7A1. The short-chain dehydrogenase/reductase superfamily is another class of aldehyde reducing enzymes responsible for the detoxification of aldehydes in cells. This class of enzymes includes carbonyl reductase (CR) and hydroxypyruvate reductase (GRHPR). CR is considered the main quinone oxidoreductase in human liver and catalyzes the two-electron reductive detoxification of quinones, including PAHs [118]. Another class of aldehyde metabolizing enzymes are GSH-dependent, including ADH5, GSTs, and glyoxalase 1 (GLO1). The class III alcohol dehydrogenase detoxifies formaldehyde via glutathione conjugation. Glutathione conjugation is catalyzed by glutathione transferases and predominantly forms conjugates with alkenals and hydroxyalkenals. Glyoxal and methylglyoxal are metabolized by glutathione conjugation and subsequent isomerization by glyoxalases [1]. The activities of these enzymes in living cells dictate the toxicity of aldehydes. Given these well-established associations of reactive carbonyls in cellular metabolism and contributions in human diseases, methods that will allow the elucidation of their roles and functions in biological systems are needed. This panel of biomarkers could be used to determine exposure, early disease diagnosis, and for monitoring disease progression, as well as therapeutic efficacy.

## 4. Bioanalytical and Mass Spectrometric Methods for Characterizing Aldehydes

There are a wide variety of analytical and biochemical techniques used to identify and quantify aldehydes. Traditionally, the analysis of aldehydes or carbonyl compounds is performed on matrices such as air, water, and soil for environmental monitoring of air and water quality by US federal agencies such as the US EPA, NIOSH, and ASTM (see Section 4.2 below) [119,120,121,122,123]. Because aldehydes play important roles in cellular processes and are linked to various diseases, these methods were further extended for the identification and characterization of these compounds in biological fluids such as plasma, cerebrospinal fluid (CSF), urine, exhaled breath condensate (EBC), and saliva. One challenging aspect in the measurement of aldehydes in biological matrices is their inherent volatility, polarity, and biochemical instability. Thus, derivatization is commonly used for the analysis of low molecular weight aldehydes in complex matrices to improve chromatographic separation, MS ionization, and MS/MS fragmentation detectability [119,124,125,126,127]. A wide range of derivatization reagents, as previously reviewed by Santa [124], and analytical methods are being applied for the analysis of carbonyl compounds in food and beverages, as previously reviewed by Osorio [39]. The different derivatization techniques and analytical methods used to identify and measure these compounds have their strengths and limitations, and, depending on the information one wants to obtain, there are techniques and experimental strategies that are suitable for each specific application. Nonetheless, methods to improve the overall sensitivity and detection of aldehydes in complex biological matrices are still being developed to enable trace level analysis and allow elucidation of their contributions and impact on human health.

### 4.1. Colorimetric/Fluorimetric/Amperometric Methods

**Figure 3:**
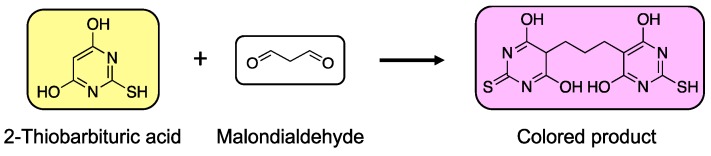
Reaction of 2-thiobarbituric acid (2-TBA) with malondialdehyde (MDA), a biomarker of oxidative stress. 2-TBA reacts with MDA to form a colored product, which is measured spectrophotometrically at 532 nm. The intensity of the colored product reflects the level of lipid peroxidation in the sample.

One of the most commonly used methods for the analysis of aldehydes in biological fluids is the assay of thiobarbituric acid reactive substances (TBARS), which are produced under high oxidative stress conditions resulting from lipid peroxidation. Oxidation of lipids generates reactive and unstable lipid hydroperoxides and further decomposition of these hydroperoxides yields MDA, a well-known biomarker of oxidative stress. MDA forms a 1:2 adduct with 2-thiobarbituric acid (2-TBA) and can be measured spectrophotometrically or fluorimetrically [128,129] (Figure 3). Although the specificity of this approach is in question as TBA can react with compounds other than MDA, it is still widely applied to measure lipid peroxidation in various biological samples including animal and human tissues and biofluids, as well as food and drugs [129]. One strategy employed to overcome the limitation of this assay is the prior precipitation of lipoproteins to eliminate interfering soluble 2-TBA-reactive substances. As TBARS are minimized, the assay becomes quite specific for lipid peroxidation [129,130]. In addition, extraction of MDA-reactant adducts is also employed, however, this approach introduces another time-consuming step and adversely affects precision of the assay [130].

**Figure 4:**
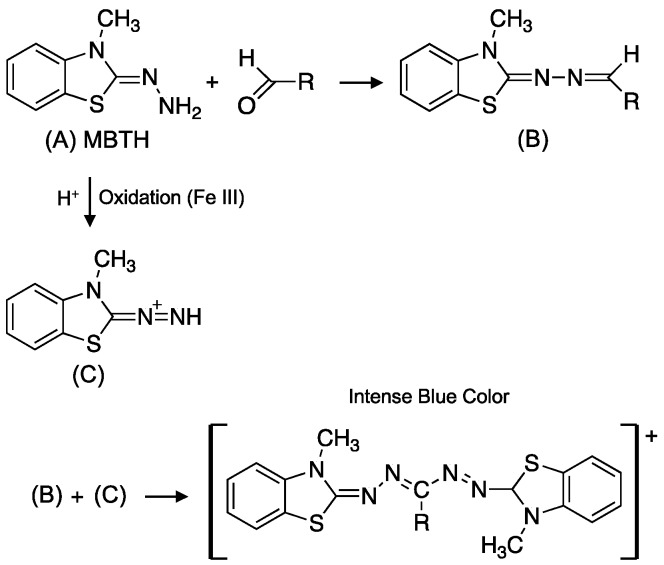
Reaction of MBTH with aldehydes to form an intense blue-colored complex. Figure adapted from Reference [131] (Copyright 2016, Elsevier).

Another rapid and simple strategy to determine aldehydes in biological fluids, such as saliva, is the development of a microfluidic paper-based analytical device (μPAD) [131]. This device is based on the reaction of aldehydes with 3-methyl-2-benzothiazolinone hydrazine (MBTH) and iron (III) to form a blue formazan complex, which can be evaluated visually (Figure 4) [131]. This approach is simple, rapid, and non-invasive for the analysis of salivary aldehydes, which could be useful in assessing oral cancer risk in population-based studies and point-of-care diagnostics for aldehyde exposure. Methods based on capillary electrophoresis, coupled with amperometric detection (CE-AD) and using electroactive 2-TBA, have been developed and used to analyze two non-electroactive aldehydes, methylglyoxal and glyoxal in urine and water samples. This method demonstrates good specificity for methylglyoxal and glyoxal with the formation of stable pink-chromophore adducts with 2-TBA. Using this approach, the LODs (limit of detection) obtained are 0.2 μg L^−1^ (0.6 nmol L^−1^) and 1.0 μg L^−1^ (3.2 nmol L^−1^) for methylglyoxal and glyoxal, respectively [132]. The approaches described above are simple and the instrumentation is easy to use and operate for rapid screening of aldehydes in various matrices. In addition, these analytical techniques can be applied for remote monitoring of aldehydes where more sophisticated bioanalytical tools and mass spectrometry instrumentation are not available. The limitations of these techniques, however, are their low specificity and selectivity for identifying aldehydes, which can be further confounded with increased matrix complexity.

### 4.2. High-Performance Liquid Chromatography (HPLC) with Ultraviolet (UV)/Fluorescence Detection

Historically, HPLC-UV has been the method of choice for characterizing and quantifying aldehydes in a wide array of matrices and were originally developed for environmental analysis. However, characterization and quantification of aldehydes has gained widespread use in the food and beverage industry, and in the biomedical field, where aldehydes have been shown to play major roles in cellular processes and disease pathogenesis. In addition, the derivatization of carbonyl compounds is typically accomplished using 2,4-dinitrophenylhydrazine (DNPH) to form their corresponding carbonyl-hydrazones. The carbonyl-hydrazones are then analyzed by HPLC with ultraviolet detection. HPLC-UV detection is commonly used to characterize and quantify carbonyl compounds in various matrices because of its simplicity, robustness, and reproducibility. DNPH derivatization and HPLC-UV analysis are used in environmental monitoring of air and water quality and used for screening and monitoring carbonyl compounds in various matrices by the US federal agencies (Table 1) [119,133,134,135,136,137]. The HPLC-UV technique is also being used in the food industry to measure aldehydes in food and beverages [39,138,139,140,141,142] and in biomedical research to measure aldehydes and carbonyls in various matrices such as urine, plasma and serum samples [40,143,144,145,146,147,148,149,150,151,152]. DNPH derivatization is also used in conjunction with a reducing agent, 2-picoline borane (2-PB) to stabilize carbonyl-hydrazones and to resolve isomeric compounds produced during the reaction that might interfere with subsequent quantitative analysis by HPLC-UV [153]. DNPH and hydroquinone impregnated into silica cartridges has been used for the determination of acrolein and other carbonyl compounds in cigarette smoke [22]. This approach is useful for characterizing carbonyls in air samples for environmental analysis as well as for the characterization of other α,β-unsaturated aldehydes in tobacco smoke. DNPH derivatization was also used for the analysis and measurement of acetaldehyde in plasma and red blood cells [154], formaldehyde determination in human tissue [151], carbonyl compounds in exhaled breath of e-cigarette users [35], and for the measurement of formaldehyde released from heated hair straightening cosmetic products [18]. Other reagents such as the previously mentioned 2-thiobarbituric acid (2-TBA) and diaminonapththalene (DAN) are also being used for HPLC-UV analysis of carbonyl compounds from biological matrices and environmental samples [155,156,157].

To improve sensitivity and allow for simultaneous derivatization and extraction of derivatized carbonyls for HPLC-UV analysis, a wide array of sample preparation techniques have been introduced into the analytical workflows. For instance, a method for the quantification of early lung cancer biomarkers, hexanal and heptanal in urine, has been developed using a bar adsorptive microextraction (BAμE) technique and DNPH derivatization. This approach uses an adsorptive bar impregnated with the derivatization reagent for simultaneous derivatization and extraction of derivatized carbonyls. The LODs obtained for hexanal and heptanal are 0.80 μmol L^−1^ (800 nmol L^−1^) and 0.40 μmol L^−1^ (400 nmol L^−1^), respectively [145]. Similarly, magnetic solid phase extraction coupled with in-situ DNPH derivatization (MSPE-ISD) was developed for the determination of hexanal and heptanal in urine. The extraction, purification, and derivatization of aldehydes are integrated into a single analytical step, simplifying the measurement workflow. The LODs are 1.7 and 2.5 nmol L^−1^ for hexanal and heptanal, respectively. Using this approach, the levels of hexanal and heptanal in urine of lung cancer patients were found to be higher compared to healthy controls [147]. Another method for the analysis of hexanal and heptanal in plasma used DNPH adsorbed on a polymer monolith composed of poly(methacrylic acid-co-ethylene glycol dimethacrylate) for simultaneous derivatization and microextraction, followed by HPLC-UV analysis. The LODs obtained are 2.4 and 3.6 nmol L^−1^ for hexanal and heptanal, respectively [150]. This monolith microextraction technique was further extended and used for the analysis of 5-hydroxymethylfurfural (5-HMF) in beverages such as coffee, honey, beer, soda, and urine [142]. In addition, a method using dispersive liquid–liquid microextraction with 1-dodecanol of DNPH derivatized aldehydes has been developed. Centrifugation of the sample and subsequent solidification of the droplet on an ice bath for easy removal of derivatized compounds for HPLC-UV analysis was performed. The LODs obtained for hexanal and heptanal are 7.90 nmol L^−1^ and 2.34 nmol L^−1^, respectively. This approach afforded higher sensitivity compared to the conventional liquid-liquid microextraction methods [146]. An alternative approach developed by the same group uses ultrasound-assisted headspace liquid-phase microextraction with in-drop derivatization for the extraction and determination of hexanal and heptanal in blood. This technique uses a polychloroprene PCR tube containing the extraction solvent, methyl cyanide and the derivatization reagent, DNPH. Volatile aldehydes are then headspace extracted and derivatized simultaneously in the droplet and analyzed by HPLC-UV. The LODs for hexanal and heptanal are 0.79 nmol L^−1^ and 0.80 nmol L^−1^, respectively [148].

In addition to UV detection, fluorogenic derivatization reagents for the HPLC analysis of aldehydes are widespread in the literature. These tagging reagents are used either as pre-column labeling reagents or in one-pot derivatization of aldehydes. For instance, the labeling reagent 1,3,5,7-tetramethyl-8-aminozide-difluoroboradiaza-s-indacence (BODIPY-aminozide) is used as a pre-column derivatization reagent to monitor aldehydes in human serum by HPLC with fluorescence detection [158]. The BODIPY-based reagent reacts with aldehydes to form stable and highly fluorescent BODIPY hydrazone derivatives, which are easily separated and detected by HPLC with fluorescence detection at 495 nm (maximum excitation wavelength) and 505 nm (maximum emission wavelength). This approach is used to measure trace aliphatic aldehydes in serum samples without pretreatment or enrichment method [158]. Other reagents used for pre-column labeling are 2,2′-furil to label aldehydes [159] and 4-(*N,N*-dimethylaminosulfonyl)-7-hydrazino-2,1,3-benzoxadiazole to label 4-HNE in human serum [160]. For the one-pot-derivatization of aldehydes, rhodamine B hydrazide (RBH) [161], 2-aminoacridone [162], 9-fluorenylmethoxycarbonyl hydrazine (FMOC-hydrazine) [163], and 2-TBA [164] are used for the determination of malondialdehyde in biological fluids [161] by HPLC with fluorescence detection. For the determination of methylglyoxal, glyoxal, and diacetyl using HPLC-fluorescence, the most commonly used derivatization reagents are 4-methoxy-*o*-phenylenediamine (4-MPD) [165] and 1,2-diamino-4,5-dimethoxybenzene (DDB) [166]. Monitoring of methylglyoxal and glyoxal in diabetic patients has been proposed to help assess the risk of development of diabetic complications. Additionally, an increase in oxidative stress biomarkers has been reported in juvenile swimmers but no prior data has been reported on α-ketoaldehydes in urine associated with swim training. Thus, these methods were applied to compare the levels of these molecules in urine samples from healthy volunteers, diabetic subjects, and juvenile swimmers [165]. For acrolein analysis, luminarin 3 [167] and *m*-aminophenol [168] were used for the derivatization and HPLC-fluorimetric analysis in plasma resulting from the metabolism of drugs such as cyclophosphamide and ifosfamide [167]. HPLC coupled with UV or fluorescence detection are widely used techniques for aldehyde analysis in various environmental and biological matrices. These techniques have been the methods of choice as they offer good sensitivity and robustness. Along with innovative sample pre-treatment incorporated into the assays, low detection limits were obtained for quantifying specific biomarkers associated with various diseases. However, these methods do not provide structural information relating to the analyte of interest and require synthetic standards for analyte identification and confirmation. Finally, co-eluting peaks during HPLC separation can further confound the identification and quantitation of known and unknown carbonyl compounds via UV or fluorescence.

### 4.3. Aldehyde Visualization in Cells

**Figure 5:**
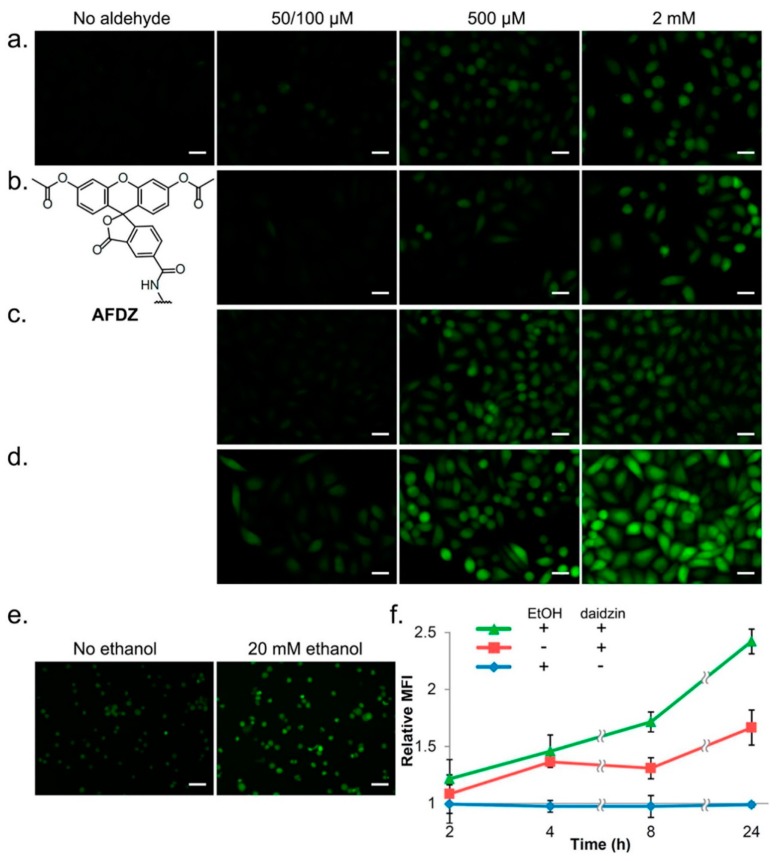
Real-time imaging of total aldehydic load in cells. Cellular aldehyde labeling fluorescence images and flow cytometry data. Hela cells were exposed to varying concentrations of: (**a**) formaldehyde; (**b**) glycolaldehyde; (**c**) acrolein; and (**d**) acetaldehyde along with 20 μM of the dye AFDZ and 10 mM catalyst (2,4-dimethoxyaniline) with images taken after 1 h of incubation. Note that 50 μM was used with acrolein and 100 μM for the other aldehydes tested. (**e**) K562 cells pretreated with 250 μM daidzin and incubated with 40 μM of AFDZ dye, 10 mM catalyst (2,4-dimethoxyaniline), and with/without 20 mM ethanol. (**f**) Flow cytometry data monitoring the production of aldehyde over time in K562 cells with/without ethanol. The fluorescence intensities were compared to that obtained from *t* = 0 without added ethanol and daidzin. Scale bars (20 μM) are shown. Reprinted from [171] (Copyright 2016, American Chemical Society).

In addition to HPLC with fluorimetric detection, fluorescent probes were designed and synthesized for real-time visualization of aldehydes in cells such as FP1 and FAP-1 for formaldehyde detection [169,170]. These formaldehyde probes are based on the 2-aza-Cope sigmatropic rearrangement, which yields highly fluorescent signal for the selective and sensitive detection of aldehydes in cells [169,170]. Recently, a novel technique based on real-time imaging of aldehydes in cells using multicolor fluorogenic hydrazone transfer (“DarkZone”) was developed (Figure 5). This approach used a cell permeable DarkZone dye (7-(diethylamino)coumarin; DEAC) as a quenched hydrazone, which lights up when the quencher-aldehyde is replaced by the target aldehyde. The fluorescence signals are then detected by flow cytometry or microscopy without the need for washing or cell lysis. This strategy is useful for determining the aldehyde load associated with human diseases [171]. Recently, a novel fluorescent probe to visualize specific and total biogenic carbonyls was developed based on the pattern and fluorescence spectral profile unique to the target carbonyl compound. The probe is based on an *N*-aminoanthranilate methyl ester moiety [96]. These techniques offer real time monitoring of total aldehydes in cells and identification of specific aldehydes based on their unique fluorescence excitation and emission spectra. Overall, real-time imaging of aldehyde production in cells using aldehyde-specific probes allows elucidation of the roles and functions of these compounds in cellular processes and their involvement in disease pathogenesis. These techniques, however, lack the selectivity and specificity for the identification of specific carbonyls in cells as no structural information can be obtained. Finally, these techniques are not applicable to biological matrices such as blood, urine, CSF or saliva.

### 4.4. Gas Chromatography (GC)/Gas Chromatography-Mass Spectrometry (GC-MS)

Mass spectrometry is widely used for the characterization and quantification of carbonyl compounds providing more selectivity, specificity, and sensitivity than is possible with UV or fluorescence detection [39,124,172]. There are a wide variety of derivatization reagents and sample preparation methods used to enhance the detection and sensitivity for mass spectrometric analysis of aldehydes (Table 2). For GC-MS analysis, derivatization increases the volatility of aldehydes in biological fluids and is most commonly done with *O*-2,3,4,5,6-pentafluorobenzyl hydroxylamine hydrochloride (PFBHA) as has been used for the analysis of saliva-available carbonyls in chewing tobacco products [173], to measure methylglyoxal and glyoxal in plasma of diabetic patients [174], formaldehyde in urine [175], and for the determination of MDA and 4-HNE levels in plasma [176]. In addition, PFBHA derivatization is often performed using headspace microextraction with subsequent derivatization on-fiber, on droplet, or for simultaneous extraction, derivatization, and GC-MS of volatile carbonyls. For instance, a quantitative method for the analysis of hexanal, heptanal, and volatile aldehydes in human blood was developed using headspace solid-phase microextraction with on-fiber derivatization with PFBHA and subsequent analysis by GC-MS. This approach afforded LODs of 0.006 nM (0.006 nmol L^−1^) and 0.005 nM (0.005 nmol L^−1^) for hexanal and heptanal, respectively [177,178]. Similarly, this approach is implemented for the determination of hexanal, heptanal, octanal, nonanal, and decanal in exhaled breath [179,180] and for the analysis of volatile low molecular weight carbonyls in urine [181]. Likewise, several volatile organic compounds (C3–C9 aldehydes) as promising biomarkers of non-small cell lung cancer (NSCLC) are identified in exhaled breath of patients with lung cancer using on-fiber-derivatization with PFBHA. The LOD and LOQ obtained for all aldehydes are 0.001 nM and 0.003 nM, respectively [182]. On-fiber derivatization using 2,2,2-trifluoroethylhydrazine (TFEH) as derivatization reagent is also used for the analysis of MDA in blood [183].

In addition, PFBHA derivatization on droplet is used for the analysis of hexanal and heptanal in blood [184]. This strategy involves the dissolution of the derivatizing agent in an organic solvent such as decane, and volatile aldehydes are headspace extracted and derivatized in the droplet with subsequent injection for GC-MS analysis. Likewise, a stir bar sorptive extraction (SBSE) for the GC-MS analysis of 4-HNE in urine was developed. This approach used a stir bar impregnated with the derivatization agent, PFBHA. The resulting oximes were further acylated using sulfuric acid and thermally desorbed and analyzed by GC-MS. This approach affords LOD of 22.5 pg mL^−1^ (0.06 nmol L^−1^) and LOQ of 75 pg mL^−1^ (0.19 nmol L^−1^) for the target carbonyl, 4-HNE [185]. PFBHA is also used in combination with other derivatization reagents. For example, a novel two-step derivatization approach using PFBHA as the first derivatizing agent followed by *N*-Methyl-*N*-trimethylsilyl-trifluoroacetamide (MSTFA) was developed for the analysis of glyoxal, methylglyoxal, and 3-deoxyglucosone in human plasma by GC-MS [186]. Other derivatization reagents used for GC-MS are 2,3,4,5,6-pentafluorobenzyl bromide (PFB-Br) [187,188] and 2,4,6-trichlorophenylhydrazine (TCPH) [189] for the analysis of MDA in urine; phenylhydrazine (PH) for the analysis of MDA in plasma and rat liver microsomes [190]; pentafluorophenyl hydrazine (PFPH) for the analysis of carbonyls in MTS [23]; 2,3-diaminonaphthalene along with salting-out assisted liquid–liquid extraction (SALLE) and dispersive liquid–liquid microextraction (DLLME) for the analysis of glyoxal and methylglyoxal in urine [191]; and meso-stilbenediamine [192] and 1,2-diaminopropane [193] for the analysis of methylglyoxal serum of diabetic patients and healthy controls by capillary GC-FID.

Methods based on gas chromatography without prior derivatization are also used for the analysis of volatile aldehydes. For example, a GC-MS coupled to a headspace generation autosampler is used for the analysis of endogenous aldehydes in urine as potential biomarkers of oxidative stress [194] and carbonyls such as acetaldehyde, propionaldehyde, acrolein, and crotonaldehyde in MTS [195]. Similarly, acetaldehyde in saliva of subjects after alcohol consumption is determined without prior derivatization using headspace extraction and GC coupled with flame ionization detector (FID) [40]. No prior derivatization is also applied to characterize toxic compounds such as benzene, toluene, butyraldehyde, benzaldehyde, and tolualdehyde in saliva using micro-solid-phase extraction (μSPE) and GC-IMS [196]. Gas chromatography coupled with various detection systems such as FID and mass spectrometry are ideal tools in the direct analyses of volatile carbonyl compounds in complex matrices. These techniques are useful for low molecular weight, volatile aldehydes. However, these methods require derivatization for the analysis of high-molecular weight, less volatile carbonyls.

### 4.5. Liquid Chromatography-Mass Spectrometry (LC-MS)

#### 4.5.1. Methods Based on Selected Reaction Monitoring (SRM)

**Figure 6:**
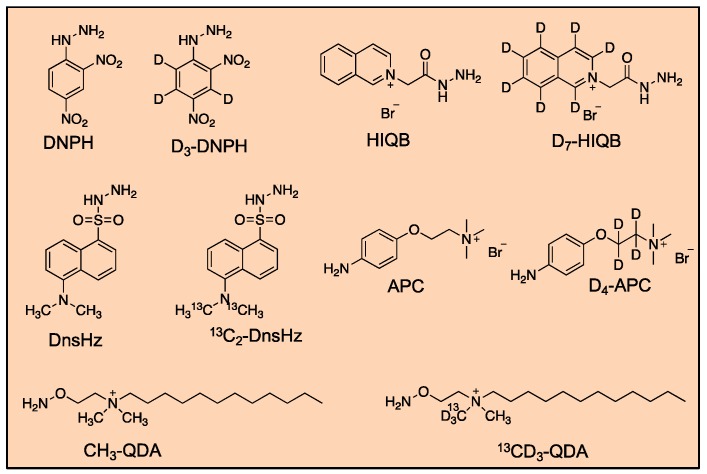
Commonly used differential isotope labeling reagents for profiling and relative quantitation of carbonyl compounds.

Liquid chromatography–mass spectrometry-based approaches have been used extensively to quantify derivatized carbonyl compounds, and recently for screening of unknown carbonyl compounds. Aldehyde derivatizations using 2,4-DNPH [143,197,198,199,200,201,202], dansylhydrazine (DnsHz) [203,204], *N*-(1-chloroalkyl)pyridinium [205], *o*-phenyldiamine [206], D-cysteine [207], 9,10-phenanthrenequinone (PQ) [208], 3-nitrophenylhydrazine [209], and 3,4-diaminobenzophenone [210] have been used to provide chromatographic retention and separation, efficient MS ionization, and MS/MS detectability. Typically, LC-MS analysis has been performed using selected reaction monitoring (SRM) with either atmospheric pressure chemical ionization (APCI), atmospheric pressure photoionization (APPI) or electrospray ionization (ESI). For example, D-cysteine has been used to generate alkyl-thiazolidine-carboxylic acid derivatives and analyzed by LC-SRM to quantify aldehydes in beverages with an LOD and LOQ of 0.2–1.9 μg L^−1^ (1.36–8.76 nmol L^−1^) and 0.7–6.0 μg L^−1^ (4.76–27.6 nmol L^−1^), respectively [207]. Alternatively, a method for profiling lipophilic reactive carbonyls in biological samples based on dansylhydrazine derivatization and LC-SRM has been developed with monitoring of the characteristic product ion, *m/z* 236.1 corresponding to 5-dimethylaminonaphthalene-1-sulfonyl moiety. This approach detects 400 free reactive carbonyls in plasma samples from mice, of which 34 are confirmed by synthetic standards [204]. Furthermore, charged derivatization reagents, such as 4-(2-(trimethylammonio) ethoxy) benzenaminium halide (4-APC), 4-(2-((4-bromophenethyl)dimethylammonio)ethoxy)benzenaminium dibromide (4-APEBA), N-[2-(aminooxy)ethyl]-N,N-dimethyl-1-dodecylammonium (QDA), and *N,N,N*-triethyl-2-hydrazinyl-2-oxoethanaminium bromide (HIQB), have been used to enhance ionization of the carbonyls for LC-MS analysis. For example, 4-APC, which contains an aniline moiety for reaction with aliphatic aldehydes, and a quaternary ammonium group for improved ionization efficiency and sensitivity, was developed for the analysis and quantitation of aldehydes in biological fluids [211] (Figure 6). Similarly, a second-generation derivatization reagent, 4-APEBA, consisting of a bromophenethyl group for isotopic signature incorporation and additional fragmentation identifiers, has been developed [212]. Another labeling reagent using *N*-(1-chloroalkyl)pyridinium quaternization to provide a charged tag was developed for quantifying aliphatic fatty aldehydes. This approach is used to measure the levels of long-chain non-volatile fatty acids in thyroid carcinoma tissues [205].

Assays with simultaneous derivatization and analysis have been developed. For example, a fully automated in-tube solid phase microextraction/liquid chromatography-post column derivatization with hydroxylamine hydrochloride and mass spectrometry was developed for the analysis of hexanal and heptanal in human urine as potential biomarkers for lung cancer [213]. In addition, this approach has been extended to the analysis of urinary malondialdehyde by DNPH derivatization and LC-SRM [198]. Similarly, an approach based on magnetic solid phase extraction coupled with in-situ derivatization with 2,4-DNPH was developed for the determination of hexanal and heptanal in urine of lung cancer patients [147]. Likewise, an Alternate Isotope-Coded Derivatization (AIDA) was developed to quantify malondialdehyde and 4-HNE in exhaled breath condensate by LC-SRM. This approach affords good quantitation of MDA and 4-HNE and is in good agreement with quantitation of the same samples using external calibration [199].

#### 4.5.2. Screening LC-MS Methods

SRM analysis provides excellent sensitivity and good specificity for quantitative analysis but lacks the ability to screen for unknown aldehydes and requires a knowledge of unique SRM transitions of the known carbonyl compounds to be measured. Thus, data-dependent LC-MS/MS analysis (DDA) with DNPH derivatization is frequently used for untargeted profiling with MS^n^ spectra used for identification and structural elucidation [135,215,229]. Studies using negative ionization have described the MS and MS/MS behavior of DNPH-derivatized carbonyls [215,216,229]. Studies using positive electrospray ionization have characterized DNPH-derivatized malondialdehyde [198,199,217,230] and 4-HNE [199], and recently we characterized the positive ionization and fragmentation of a wide range of DNPH-derivatized carbonyls to establish consistent fragmentation rules applicable to this class of compounds, allowing for screening of unknown carbonyl compounds and comprehensive detection [218] (Table 3).

##### Differential Isotope Labeling for Profiling and Relative Quantitation of Aldehydes

To allow simultaneous identification and quantitation of carbonyl compounds in biological fluids and alcoholic beverages, isotopically labeled counterparts are used for differential labeling (Figure 6). 4-APC and its labeled counterpart, D_4_-4-APC, have been used for untargeted profiling of aldehydes by differential stable isotope labeling using liquid chromatography-double neutral loss scan-mass spectrometry (SIL-LC-DNLS-MS). Pooled control samples are labeled with isotope labeled compounds, while the individual samples are derivatized with the unlabeled versions. This approach involves scanning of the two characteristic neutral fragments of 87 Da and 91 Da generated upon CID corresponding to the unlabeled 4-APC and labeled D_4_-4-APC-derivatized carbonyls, respectively. This strategy enables profiling of 16 and 19 aldehyde-containing compounds in human urine and white wine, respectively. Finally, five aldehydes in human urine and four aldehydes in white wine are confirmed by comparison with synthetic standards [219]. This approach was further extended using an enrichment step by solid phase-extraction using stable isotope labeling–solid phase extraction–liquid chromatography–double precursor ion scan/double neutral loss scan–mass spectrometry analysis (SIL-SPE-LC-DPIS/DNLS-MS) for profiling and relative quantitation of aldehydes in beer. The pair of isotope reagents, 4-APC and D_4_-4-APC, are used for differential labeling of the samples and co-eluting *m/z* pairs separated by 4 Da were detected and identified in the mass spectral data obtained by high resolution LC-QTOF-MS. Using this approach, 25 candidate aldehydes are detected in beer. The 25 candidate aldehydes are then quantified in different beer samples using a targeted MRM approach by monitoring the MRM transitions [M]^+^ → [M]^+^ − 87 and [M+4]^+^ → [M + 4]^+^ − 91 corresponding to 4-APC and D_4_-4-APC, respectively. Fifteen aldehydes are identified and confirmed by comparison with synthetic standards and MS/MS analysis [220]. Likewise, differential labeling for profiling and relative quantitation of fatty aldehydes in biological samples using 2,4-bis-(diethylamino)-6-hydrazino-1,3,5-triazine and its deuterated counterpart has been developed. Using the 2VO dementia rat model system, 43 and 19 fatty aldehydes are significantly altered between the controls and models groups’ plasma and brain tissue, respectively [214].

A high-performance chemical isotope labeling (CIL)-LC-MS method for profiling and quantitative analysis of carbonyl sub-metabolome in human urine using dansylhydrazine (DnsHz) as labeling reagent has been developed [222]. Identification and relative quantitation of carbonyl metabolites was performed using differential tagging with ^12^C-DnsHz and ^13^C-DnsHz in urine samples and subsequent analysis using LC-QTOF-MS. In-house software program was developed to process the CIL LC-MS mass spectral and a custom library of DnsHz-labeled standards was constructed (www.mycompoundid.org) for carbonyl metabolites identification. In total, 1737 peak pairs are detected in human urine, of which 33 are confirmed [222]. In addition, a strategy based on isotope labeling and liquid chromatography–double precursor ion scan mass spectrometry (IL-LC-DPIS-MS) was developed for the comprehensive profiling and relative quantitation of carbonyl compounds in human serum using the labeling reagent, HIQB and its corresponding isotope-labeled analog, D_7_-HIQB [222]. The characteristic products ions, *m/z* 130.1/137.1 are monitored in the double precursor ion scans during mass spectrometry analysis upon collision-induced dissociation (CID). In total, 156 candidate carbonyl compounds are detected in human serum, of which 12 are further identified by synthetic standards. Using a targeted MRM mode, 44 carbonyls are found to be statistically different in myelogenous leukemia patients compared to healthy controls [223].

##### Methods Using High-Resolution/Accurate Mass Data Dependent Acquisition (DDA) and Data Independent Acquisition (DIA)

**Figure 7:**
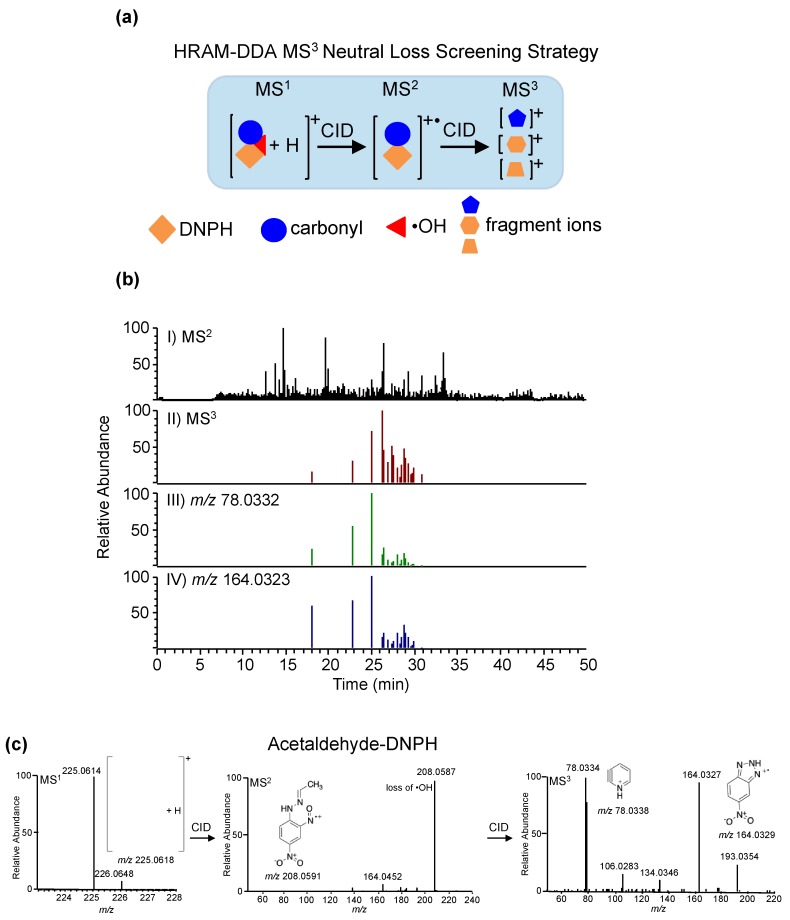
Development of a high-resolution accurate mass data-dependent MS^3^ neutral loss screening strategy for profiling and quantitative analysis of aldehydes in biological fluids. (**a**) The high-resolution accurate mass of ^•^OH (17.0027 Da) was used to screen for all DNPH-derivatized aldehydes. (**b**) Monitoring of specific fragment ions (*m/z* 78.0332 and *m/z* 164.0323) minimizes possible false positive identification. (**c**) Representative MS, MS^2^, and MS^3^ spectra of DNPH-derivatized acetaldehyde and proposed structures of major fragment ions. Reprinted with permission from Ref. [218] (Copyright 2017, Springer).

High-resolution mass spectrometry-based methods for metabolomics profiling provide accurate masses of both precursor and MS/MS fragment ions, and thus allow confident identification of detected metabolites in complex biological matrices. Recently, we have developed a high-resolution accurate mass data-dependent MS^3^ neutral loss (NL) screening strategy to characterize DNPH-derivatized carbonyls in biological fluids, allowing for the simultaneous detection and quantitation of suspected and unknown/unanticipated carbonyl compounds [218]. Previous analyses of DNPH-derivatized carbonyls were mostly performed in negative ionization mode and at relatively high-flow rates, which limit the sensitivity of detection and quantitation of trace level analytes (Table 3). We found that, in positive mode, these compounds showed a characteristic neutral loss of hydroxyl radical (^•^OH) upon CID. This NL is not observed in negative mode. The characteristic neutral loss, ^•^OH from DNPH-derivatized carbonyls, is then used as a screening approach during MS acquisition allowing unambiguous identification of RCCs (Figure 7). Furthermore, a relative quantitation strategy by differential isotope labeling using D_0_-DNPH and D_3_-DNPH is implemented to determine the relative levels of carbonyls after specific exposures. Using this approach, pre-exposure samples are labeled with D_0_-DNPH, while post-exposure samples are labeled with D_3_-DNPH. The samples are combined in a 1:1 (v/v) ratio and analyzed by our HR-AM NL screening strategy. The MS-based workflow provides an accurate, rapid, and robust method to identify and quantify toxic carbonyls in various biological matrices for exposure risk assessment. This is in contrast to previous work, which used relatively high flow rates (0.2–1.5 mL min^−1^) and low-resolution MS analysis, limiting their sensitivity and identification confidence at trace analyte levels. We applied this method to characterize the levels of carbonyls after alcohol consumption in humans and showed that acetaldehyde levels are increased after exposure. This strategy is currently being used to characterize the carbonyls associated with e-cigarette use (vaping) as well as tobacco smoking.

Another strategy based on ultra-high-resolution fourier transform mass spectrometry (UHR FT-MS) method using the tribrid orbitrap fusion was developed for profiling carbonyl metabolites in crude biological extracts. This approach uses a chemoselective tagging reagent, QDA, and its labeled counterpart, ^13^CD_3_-QDA, for differential isotope labeling of biological samples. Data-dependent TopN MS/MS of the targeted mass difference of 4.0219 Da (QDA and ^13^CD_3_-QDA metabolite pairs) is performed with direct infusion allowing for long acquisition times, resolved isotopic peaks and high-quality MS and MS/MS data. MS and MS/MS spectral data are processed using a custom software Precalculated Exact Mass Isotopologue Search Engine (PREMISE) for QDA–^13^CD_3_-QDA ion pairs and isotopologue identification. The workflow identifies 66 carbonyls in mouse tumor tissues, of which 14 carbonyls are quantified using authentic standards [231]. A similar derivatization and differential labeling approach is applied for the profiling and untargeted metabolomics of carbonyl compounds in cell extracts [226]. Likewise, direct infusion and FT-ICR-MS are used for the analysis of aldehydes and ketones in exhaled breath using 2-(aminooxy)ethyl-*N,N,N*-trimethylammonium iodide (ATM) and 4-(2-aminooxyethyl)-morpholin-4-ium chloride (AMAH) as derivatizing agents [227,228]. ATM is chemically functionalized on a novel microreactor to selectively preconcentrate volatile aldehydes and ketones. This approach demonstrated detection of C1-C12 aldehydes and applicable to any gaseous samples [227]. Similarly, AMAH is used as derivatizing agent coated within a silicon microreactor to capture volatile carbonyls to form AMAH-carbonyl adducts and analyzed by FT-ICR-MS. Subsequent treatment of the derivatized-carbonyl adducts with poly(4-vinylpyridine) yielded volatile carbonyl adducts, which can be analyzed using GC-MS. These complementary approaches using FT-ICR-MS and GC-MS provide a convenient and flexible identification and quantification of isomeric volatile organic compounds in exhaled breath [228]. In addition, an on-line weak-cation exchange liquid chromatography–tandem mass spectrometry using the LC-QTOF-MS^2^ has been developed for screening aldehydes in plasma and urine samples. This strategy involves derivatization of aldehydes with 4-APC and subsequent reduction by NaBH_3_CN. The characteristic MS/MS fragmentation of 4-APC derivatized aldehydes allows confirmation of known aldehydes as well as differentiation of hydroxylated and non-hydroxylated aldehydes [221]. Finally, a novel DIA strategy has been developed for the global analysis of aldehydes and ketones in biological samples. The strategy is based on TSH (*p*-toluenesulfonylhydrazine) derivatization of carbonyl compounds and Sequential Window Acquisition of All Theoretical Fragment-Ion spectra (SWATH) detection. Although the TSH-derivatized carbonyls are efficiently detected in both positive and negative modes, the negative ion mode data acquisition exhibits the signature fragment ion at *m/z* 155.0172, which is monitored using ESI-QqTOF-SWATH allowing chemo-selective identification of carbonyl compounds. Using this strategy, 61 target carbonyls were successfully identified and quantified in biological samples. In addition, SWATH MS data acquisition provides high resolution accurate mass measurements of both the precursor and fragment ions, allowing for confident identification of derivatized compounds [224].

Overall, HPLC coupled with mass spectrometry techniques are powerful tools for profiling and performing quantitative analysis of aldehydes in various biological matrices. The high selectivity and specificity of these methods along with structural information obtained from MS and MS^n^ mass spectral data are ideal for identifying knowns and unknowns. The more recent LC-MS-based methods presented here offer improved sensitivity, selectivity, and specificity for the detection of aldehydes in complex biological matrices. Although these techniques are highly sensitive, they are also susceptible to matrix interferences requiring rigorous sample clean-up. In addition, these techniques require expensive instrumentation and highly trained users, and are less portable. The development of new and innovative MS-based techniques is continuously evolving towards novel applications, in particular, for trace level analysis ideal for human exposure assessment, allowing for elucidation of their contributions and impact on human health.

## 5. Future Perspectives

The increased emphasis on the need to improve methods to comprehensively characterize exposures, and the parallel development of enhanced technology is resulting in a number of exciting new analytical techniques and approaches. The introduction of the concept of the exposome, intended as the totality of chemical exposures in an individual’s life-time [232], has brought to light new analytical challenges related to the complexity of capturing the totality of various exposures, which are often chemically diverse, present in trace levels, and, in some cases, are resulting from the combination of endogenous and exogenous sources. To address this complexity, tools have been developed to analyze for specific classes of compounds resulting in a number of complementary approaches. Aldehydes are a major component of the exposome, and aldehyde exposure is important in the pathogenesis of several diseases, including certain cancers. Profiling and characterizing these compounds is particularly difficult due to their reactivity and the ubiquitous presence of many of them. The improvement of tools for the investigation of the “aldehydome”, the sum of all exogenous and endogenously-formed aldehydes, is needed to elucidate the complex roles these compounds play in physiological and pathological events. With the availability of more advanced MS instrumentation, high performance chromatographic separation, and improved bioinformatics tools, the data acquired allow for increased sensitivity, identification of specific aldehydes, and the establishment of new biomarkers of exposure and effect. Additionally, the combination of these techniques with exciting new methods for single cell detection provides the potential for detection and profiling of aldehydes at a cellular level, opening up the opportunity to minutely dissect their roles and functions in biological systems and in pathogenesis.

## Figures and Tables

**Table 1:** DNPH derivatization and HPLC-UV analysis of carbonyl compounds for environmental analysis.

Method Number	Matrix	Detection
EPA T0-11	Ambient air	HPLC-UV
EPA 8315A	Liquid, solid, and gas samples	HPLC-UV
ASTM D5197	Ambient air	HPLC-UV
NIOSH 2016 and 2532	Ambient indoor air	HPLC-UV
EPA 554	Drinking water	HPLC-UV

**Table 2:** Bioanalytical techniques for characterizing carbonyl compounds.

Analytes	Matrix	Derivatization Reagent	Analytical Method	LOD	LOQ	Reference
***Colorimetric/Fluorimetric/Amperometric***						
Malondialdehyde	Plasma	2-TBA	Fluorimetric	NR	NR	Yagi 1976 [128]
Malondialdehyde	Plasma, Serum, Tissue	2-TBA	Fluorimetric	NR	NR	Armstrong et al. 1994 [129]
Malondialdehyde	Plasma	2-TBA	Fluorimetric	0.015 μmol L^−1^	0.025 μmol L^−1^	Del Rio et al. 2003 [130]
Aldehydes	Saliva	MBTH	Colorimetric	6.1 μM	NR	Ramdzan et al. 2016 [131]
Methylglyoxal and glyoxal	Urine and water	2-TBA	CE-AD	0.2 μg L^−1^ (methylglyoxal) 0.5 μg L^−1^ (glyoxal)	1.0 μg L^−1^ (methylglyoxal) 2.0 μg L^−1^ (glyoxal)	Zhang et al. 2010 [132]
***HPLC-UV***						
Acrolein, carbonyls	Cigarette smoke	HQ/2,4-DNPH	HPLC-UV	0.015–0.074 μg	0.05–0.25 μg	Uchiyama et al. 2010 [22]
Acetaldehyde	Plasma, red blood cells	2,4-DNPH	HPLC-UV	NR	NR	Di Padova et al. 1986 [154]
Malondialdehyde	Plasma	2-TBA	HPLC-UV	0.05 μM	0.17 μM	Grotto et al. 2007 [156]
Hexanal and heptanal	Urine	2,4-DNPH	HPLC-UV	1.0 μmol L^−1^ (hexanal); 0.7 μmol L^−1^ (heptanal)	3.0 μmol L^−1^ (hexanal); 2.2 μmol L^−1^ (heptanal)	Oenning et al. 2017 [145]
Hexanal and heptanal	Blood	2,4-DNPH	HPLC-UV	7.9 nmol L^−1^ (hexanal); 2.3 nmol L^−1^ (heptanal)	NR	Lili et al. 2010 [146]
Hexanal and heptanal	Urine	2,4-DNPH	HPLC-UV	1.7 nmol L^−1^ (hexanal); 2.5 μmol L^−1^ (heptanal)	5.7 nmol L^−1^ (hexanal); 8.3 μmol L^−1^ (heptanal)	Liu et al. 2015 [147]
Malondialdehyde	Plasma	2-TBA	HPLC-UV	0.02 μmol L^−1^	NR	Nielsen et al. 1997 [155]
Malondialdehyde	Plasma, Serum	2,3-DAN	HPLC-UV	< 50 pM	NR	Steghens et al. 2001 [157]
5-Hydroxymethylfurfural	Beverages	2,4-DNPH	HPLC-UV	1.0 μg L^−1^	3.4 μg L^−1^	Wu et al. 2009 [142]
Hexanal and heptanal	Blood	2,4-DNPH	HPLC-UV	0.8 nmol L^−1^ (hexanal); 0.8 nmol L^−1^ (heptanal)	NR	Xu et al. 2010 [148]
Hexanal and heptanal	Urine and Serum	2,4-DNPH	HPLC-UV	0.8 nmol L^−1^ (hexanal); 0.8 nmol L^−1^ (heptanal)	NR	Xu et al. 2011 [149]
Hexanal and heptanal	Plasma	2,4-DNPH	HPLC-UV	2.4 nmol L^−1^ (hexanal); 3.6 μmol L^−1^ (heptanal)	NR	Zhang et al. 2007 [150]
Formaldehyde	Human Tissue	2,4-DNPH	HPLC-UV	1.5 mg L^−1^	5.0 mg L^−1^	Yilmas et al. 2016 [151]
Acetaldehyde	Cell culture media, rat blood and plasma	2,4-DNPH	HPLC-UV	> 3 μM	NR	Guan et al. 2012 [152]
Carbonyls	Air	2,4-DNPH/2-PB	HPLC-UV	NR	NR	Uchiyama et al. 2009 [153]
Carbonyls	Exhaled breath	2,4-DNPH	HPLC-UV	0.001-0.01 μg puff^−1^	NR	Samburova et al. 2018 [35]
Formaldehyde	Cosmetic products	2,4-DNPH	HPLC-UV	NR	10 mg kg^−1^	Galli et al. 2015 [18]
***HPLC-Fluorescence/Fluorescence***						
Glyoxal and methylglyoxal	Urine	DDB	HPLC-Fluorescence	NR	NR	Akira et al. 2004 [166]
Malondialdehyde	Serum, Plasma	2-TBA	HPLC-Fluorescence	NR	0.05 μmol L^−1^	Seljeskog et al. 2006 [164]
Acrolein	Urine	*m*-aminophenol	HPLC-Fluorescence	NR	NR	Al-Rawithi et al. 1993 [168]
Aliphatic aldehydes	Serum	2,2’-furil	HPLC-Fluorescence	0.19–0.50 nM	NR	Ali et al. 2013 [159]
4-HNE	Serum	DBD-H	HPLC-Fluorescence	0.06 μM	NR	Imazato et al. 2014 [160]
Malondialdehyde	Plasma, Urine	RBH	HPLC-Fluorescence	0.25 nM	0.80 nM	Li et al. 2013 [161]
Malondialdehyde	Plasma	FMOC-hydrazine	HPLC-Fluorescence	4.0 nmol L^−1^	NR	Mao et al. 2006 [163]
Glyoxal, methylglyoxal, and diacetyl	Urine	4-MPD	HPLC-Fluorescence	1.82–2.31 μg L^−1^	3.06–3.88 μg L^−1^	Ojeda et al. 2014 [165]
Acrolein	Plasma	luminarin 3	HPLC-Fluorescence	100 nM	300 nM	Paci et al. 2000 [167]
Aldehydes	Serum	BODIPY-aminozide	HPLC-Fluorescence	0.43–0.69 nM	NR	Xiong et al. 2010 [158]
Malondialdehyde	Urine	2-AA	HPLC-Fluorescence	1.8 nM	5.8 nM	Giera et al. 2011 [162]
Formaldehyde	Cells	FAP-1	Fluorescence	NR	NR	Brewer et al. 2015 [170]
Formaldehyde	Cells	FP1	Fluorescence	NR	NR	Roth et al. 2015 [169]
Total aldehydes	Cells	DarkZone dye/DEAC	Fluorescence	NR	NR	Yuen et al. 2016 [171]
Biogenic aldehydes	Aldehyde standards	methyl-5-methoxy-*N*-aminoanthranilate	Fluorescence	NR	NR	Lazurko et al. 2018 [96]
***Gas Chromatography (GC)/Gas Chromatography-Mass Spectrometry (GC-MS)***						
Methylglyoxal	Serum	1,2-diaminopropane	GC-FID	40 μg L^−1^	NR	Khuhawar et al. 2008 [193]
Methylglyoxal	Serum	meso-stilbenediamine	GC-FID	25 μg L^−1^	NR	Kandhro et al. 2008 [192]
Acetaldehyde	Saliva, blood	no derivatization	GC-FID	NR	NR	Yokohama et al. 2008 [40]
Butyraldehyde, Benzaldehyde, Tolualdehyde	Saliva	no derivatization	GC-IMS	0.38–0.49 mg L^−1^	1.26–1.66 mg L^−1^	Criado-Garcia et al. 2016 [196]
Pentanal, Hexanal, Heptanal, Octanal, Benzaldehyde	Urine	no derivatization	GC-MS	0.04–0.08 μg L^−1^	0.12–0.24 μg L^−1^	Anton et al. 2014 [194]
Acetaldehyde, propionaldehyde, acrolein, crotonaldehyde	MTS	no derivatization	GC-MS	0.014–0.12 μg cig^−1^	0.045–0.38 μg cig^−1^	Zhang et al. 2019 [195]
Volatile aldehydes	Urine	PFBHA	GC-MS	0.009–15 μg L^−1^	0.029–50 μg L^−1^	Calejo et al. 2016 [181]
Malondialdehyde	Plasma, RLM	Phenylhydrazine (PH)	GC-MS	5 pmol injection^−1^ (LLOD)	NR	Cighetti et al. 1999 [190]
Hexanal and heptanal	Blood	PFBHA	GC-MS	0.006 nM (hexanal); 0.005 nM (heptanal	NR	Deng et al. 2004 [177]
Aldehydes	Blood	PFBHA	GC-MS	0.001–0.006 nM	NR	Deng et al. 2004 [178]
Malondialdehyde	Urine	PFB-Br	GC-MS	0.7 nM	NR	Hanff et al. 2017 [187]
Glyoxal and methylglyoxal	Plasma	PFBOA	GC-MS	NR	NR	Lapolla et al. 2003 [174]
Hexanal and heptanal	Blood	PFBHA	GC-MS	0.12 nM (hexanal); 0.16 nM (heptanal	NR	Li et al. 2005 [184]
Glyoxal and methylglyoxal	Urine	2,3-DAN	GC-MS	0.12 μg L^−1^ (glyoxal); 0.06 μg L^−1^ (methylglyoxal)	0.40 μg L^−1^ (glyoxal); 0.2 μg L^−1^ (methylglyoxal)	Pastor-Belda et al. 2017 [191]
Malondialdehyde	Blood	TFEH	GC-MS	0.4 μg L^−1^	NR	Shin 2009 [183]
Malondialdehyde	Plasma, Urine	TCPH	GC-MS	0.4 μM (MSD); 0.03 μM (ECD)	NR	Stalikas et al. 2001 [189]
C6-C10 aldehydes	Exhaled breath	PFBHA	GC-MS	0.01–0.03 nM	0.02–0.1 nM	Svensson et al. 2007 [180]
Glyoxal, methylglyoxal, and 3-dG	Plasma	PFBOA; MSTFA	GC-MS	12.8–31.2 μg L^−1^	12.8–31.2 μg L^−1^	Wu et al. 2008 [186]
Formaldehyde	Urine	PFBHA	GC-MS	1.08 μg L^−1^	3.6 μg L^−1^	Takeuchi et al. 2007 [175]
Volatile aldehydes	Exhaled breath	PFBHA	GC-MS	1.3–56 pmol L^−1^	4.3–226 pmol L^−1^	Fuchs et al. 2010 [179]
Aldehydes (C3-C9)	Exhaled breath	PFBHA	GC-MS	1 x 10^-12^ M	3 x 10^-12^ M	Poli et al. 2010 [182]
4-HNE	Urine	PFBHA; sulfuric acid	GC-MS	22.5 ng L^−1^	75 ng L^−1^	Stopforth et al. 2006 [185]
Malondialdehyde and 4-HNE	Plasma	PFBHA	GC-MS	NR	NR	Tsikas et al. 2017 [176]
Carbonyls	Chewing Tobacco	PFBHA	GC-MS	NR	100-1000 ppb	Chou et al. 1994 [173]
Carbonyls	MTS	PFPH	GC-MS	NR	NR	Pang et al. 2011 [23]
Malondialdehyde	Plasma	PFB-Br	GC-MS	2 amol	200 nM (LLOQ)	Tsikas et al. 2016 [188]

NR, not reported; MTS, mainstream tobacco smoke; RLM, rat liver microsomes.

**Table 3:** LC-MS-based Methods for Characterizing Aldehydes.

Analytes	Matrix	Derivatization Reagent	Ionization Technique	Ionization Mode	Flow rate (μL min^−1^)	MS Technique	LOD	LOQ	Reference
Fatty aldehydes	Plasma, brain tissue	T3	ESI	(+)	500	LC-MS/MS	0.1–1 ng L^−1^	NR	Tie et al. 2016 [214]
Carbonyls	Air	2,4-DNPH	APCI	(−)	1000	LC-MS^n^	10 pg	NR	Kolliker et al. 1998 [215]
Carbonyls	Air	2,4-DNPH	APCI	(−)	1400	LC-MS	20–60 pg	200-600 pg	Grosjean et al. 1999 [135]
Carbonyls	Air in smog chamber	2,4-DNPH	APCI	(−)	560	LC-MS^n^	0.5–1 ng	NR	Brombacher et al. 2001 [216]
Carbonyls	Standards	2,4-DNPH	APCI	(−)	550	LC-MS/MS	2.13–30.9 pg	NR	Ochs et al. 2015 [137]
Malondialdehyde	Plasma	2,4-DNPH	ESI	(+)	400	UHPLC-HRMS	32 nM	100 nM	Mendonca et al. 2017 [217]
Carbonyls	Saliva	2,4-DNPH; D_3_-2,4-DNPH	ESI	(+)	0.3	HR/AM DDA NL MS^3^	0.19–3.24 fmol	NR	Dator et al. 2017 [218]
Carbonyls	Engine exhaust, polymers, liquid soaps	2,4-DNPH	APCI	(+) & (−)	200	LC-MS	NR	NR	Olson et al. 1985 [202]
Carbonyls	Automobile exhaust and cigarette smoke	2,4-DNPH	APPI, APCI	(−)	500	LC-MS	2.9–24 nmol L^−1^	9.7–80 nmol L^−1^	Van Leeuwen et al. 2004 [136]
Carbonyls	MTS	2,4-DNPH	ESI, APCI, APPI	(−)	500	UHPLC-MS	NR	0.022–0.134 μg mL^−1^	Miller et al. 2010 [144]
Aldehydes	EBC	2,4-DNPH	APCI	(+) & (−)	800	LC -MS/MS	0.3–1.0 nM	NR	Andreoli et al. 2003 [197]
LMM aldehydes	Urine	2,4-DNPH	ESI	(−)	300	LC-MS/MS	15–65 ng L^−1^	50–200 ng L^−1^	Banos et al. 2010 [143]
Malondialdehyde	Urine	2,4-DNPH	ESI	(+)	200	LC-MS/MS	1.6 nmol L^−1^	6.4 nmol L^−1^	Chen et al. 2011 [198]
Malondialdehyde and 4-HNE	EBC	2,4-DNPH; D_3_-2,4-DNPH	ESI	(+)	500	LC-MS/MS	NR	NR	Manini et al. 2010 [199]
Trifluoroacetaldehyde	Human liver microsomes	2,4-DNPH; D_3_-2,4-DNPH; ^15^N_4_-2,4-DNPH	ESI	(−)	200	LC-MS	16 ± 4 μg L^−1^ (SIM) 23 ± 6 μg L^−1^ (NRS) * 59 ± 32 μg L^−1^ (SRM)	NR	Prokai et al. 2012 [201]
Aldehydes and ketones	Drinking water	2,4-DNPH	ESI	(−)	300	LC-MS	25–50 pg	NR	Richardson et al. 2000 [119]
Carbonyls	Air	2,4-DNPH	ESI	(−)	600	LC-MS/MS	0.4–9.4 ng (m^3^)^−1^	NR	Chi et al. 2007 [134]
Carbonyls	Water	2,4-DNPH	ESI	(−)	300	LC-MS	0.13–0.76 μg L^−1^	0.48–2.69 μg L^−1^	Zwiener et al. 2002 [133]
Aldehydes	Cigarette smoke	2,4-DNPH	ESI	(−)	300	LC-MS	NR	NR	Van der Toorn et al. 2013 [200]
Malondialdehyde	Plasma	3-nitrophenylhydrazine	ESI	(+)	350	LC-MS/MS	0.007 μM (LLOD)	0.02 μM (LLOQ)	Sobsey et al. 2016 [209]
Malondialdehyde	Urine, saliva	3,4-diaminobenzophenone	ESI	(+)	200	LC-MS/MS	0.03–0.1 μg L^−1^	0.1–0.3 μg L^−1^	Oh et al. 2017 [210]
Aldehydes	Urine and white wine	4-APC; D_4_-4-APC	ESI	(+)	200	SIL-LC-DNLS-MS	1.2–10 nmol L^−1^	NR	Yu et al. 2015 [219]
Aldehydes	Beverages	4-APC; D_4_-4-APC	ESI	(+)	200	LC-DPIS/DNLS-MS	NR	NR	Zheng et al. 2017 [220]
Aldehydes	Plasma	4-APC; NaBH_3_CN	ESI	(+)	150	LC-MS/MS	0.5–2.5 nM	NR	Eggink et al. 2009 [221]
Aliphatic aldehydes	Urine	4-APC; NaBH_3_CN	ESI	(+)	150	LC-MS	3–33 nM	NR	Eggink et al. 2008 [211]
Aldehydes	Plasma, Urine	4-APEBA; NaBH_3_CN	ESI	(+)	150	LC-MS/MS	NR	NR	Eggink et al. 2010 [212]
Aldehydes	Beverages	4-HBA	ESI	(+)	500	LC-MS	NR	NR	De Lima et al. 2018 [141]
Aldehydes	Serum	9,10-PQ	ESI	(+)	500	LC-MS/MS	0.004–0.1 nM	0.05–0.25 nM	El-Maghrabey et al. 2016 [208]
Aldehydes	Beverages	D-cysteine	ESI	(+)	200	LC-MS/MS	0.2–1.9 μg L^−1^	0.7–6.0 μg L^−1^	Kim et al. 2011 [207]
Aldehydes	Synthesis	DAABD-MHz	ESI	(+)	200	LC-MS/MS	30–60 fmol	NR	Santa et al. 2008 [125]
Malondialdehyde	plasma	dansylhydrazine (DnsHz)	ESI	(+)	300-1500	LC-MS/MS	0.016 mg L^−1^	0.054 mg L^−1^	Lord et al. 2009 [203]
Carbonyls	Plasma	DnsHz	ESI	(+)	200	LC-MS/MS	1-20 fmol	2.5-50 fmol	Tomono et al. 2015 [204]
Carbonyls	Urine	DnsHz; ^13^C_2_-DnsHz	ESI	(+)	180	LC-MS	NR	NR	Zhao et al. 2017 [222]
Carbonyls	Serum	HIQB; D_7_-HIQB	ESI	(+)	200	IL-LC-DPIS-MS	0.1–0.21 fmol	NR	Guo et al. 2017 [223]
Aldehydes and ketones	Urine, plasma	HTMOB	ESI	(+)	Infusion	LC-MS/MS	NR	NR	Johnson 2007 [126]
Hexanal and heptanal	Urine	hydroxylamine hydrochloride	ESI	NR	200	UHPLC-MS/MS	15 nM (hexanal); 9 nM (heptanal)	NR	Chen et al. 2017 [213]
Fatty aldehydes	Tissue	*N*-(1-chloroalkyl)pyridinium	ESI	(+)	300	LC-MS/MS	< 0.3 ng L^−1^	NR	Cao et al. 2016 [205]
α-dicarbonyls	Plasma	*o*-phenyldiamine	ESI	(+)	1000	LC-MS/MS	0.5–42.2 nmol L^−1^	1.5–126.6 nmol L^−1^	Henning et al. 2014 [206]
Aldehydes and ketones	Yeast extract	*p*-toluenesulfonylhydrazine	ESI	(+) & (−)	350	SWATH-QqTOF	0.31 μM (ESI + only) 0.36 μM (ESI − only) 0.19 μM (ESI+ or ESI−)	NR	Siegel et al. 2014 [224]
Carbonyls	Tissue	QDA; ^13^CD_3_ labeled QDA	ESI	(+)	Infusion	UHR-FT-MS	0.07–0.66 nM	0.2–1.99 nM	Deng et al. 2018 [225]
Carbonyls	Cell extract	QDA; ^13^CD_3_ labeled QDA	ESI	(+)	Infusion	FT-ICR-MS	NR	NR	Mattingly et al. 2012 [226]
Carbonyls	Exhaled breath	ATM	ESI	(+)	Infusion	FT-ICR-MS	NR	NR	Fu et al. 2011 [227]
Carbonyls	Exhaled breath	AMAH	ESI	(+)	Infusion	FT-ICR-MS	NR	NR	Knipp et al. 2015 [228]
Aldehydes and ketones	Synthesis	TMPP-AcPFP; TMPP-PrG	ESI	(+)	500	LC-MS/MS	NR	NR	Barry et al. 2003 [127]

NR, not reported; * NRS, narrow range scans; EBC, Exhaled breath condensate; MTS, mainstream tobacco smoke; LMM, low molecular mass.

## Abbreviations

2,4-DNPH	2,4-dinitrophenylhydrazine
PFPH	pentafluorophenyl hydrazine
2-PB	2-picoline borane
4-APC	4-(2-(trimethylammonio)ethoxy)benzenaminium halide
4-APEBA	4-(2-((4-bromophenethyl)dimethylammonio)ethoxy)benzenaminium dibromide
HIQB	*N*,*N*,*N*-triethyl-2-hydrazinyl-2-oxoethanaminium bromide
QDA	*N*-[2-(aminooxy)ethyl]-*N*,*N*-dimethyl-1-dodecylammonium
TSH	*p*-toluenesulfonylhydrazine
PFB-Br	2,3,4,5,6-pentafluorobenzyl bromide
HTMOB	4-hydrazino-*N*,*N*,*N*-trimethyl-4-oxobutanaminium iodide
DBD-H	4-(*N*,*N*-dimethylaminosulfonyl)-7-hydrazino-2,1,3-benzoxadiazole
4-MPD	4-methoxy-*o*-phenylenediamine
FMOC-hydrazine	9-fluorenylmethoxycarbonyl hydrazine
PFBHA/PFBOA	*o*-2,3,4,5,6-pentafluorobenzyl)hydroxylamine hydrochloride
ATM	2-(aminooxy)ethyl-*N,N,N*-trimethylammonium iodide
AMAH	4-(2-aminooxyethyl)-morpholin-4-ium chloride
TBARS	thiobarbituric acid reactive substances
2-TBA	2-thiobarbituric acid
TFEH	2,2,2-trifluoroethylhydrazine
FAP-1/FP 1	formaldehyde probe 1
DEAC	diethylaminocoumarin
DAN	diaminonapththalene
RBH	rhodamine B hydrazide
DDB	1,2-diamino-4,5-dimethoxybenzene
MSTFA	*N*-methyl-*N*-trimethylsilyl-trifluoroacetamide
2-AA	2-aminoacridone
3-dG	3-deoxyglucosone
BODIPY aminozide	1,3,5,7-tetramethyl-8-aminozide-difluoroboradiaza-*s*-indacence
5-HMF	5-hydroxymethylfurfural
4-HBA	4-hydrazinobenzoic acid
DnsHz	dansylhydrazine
9,10-PQ	9,10-phenanthrenequinone
T3	2,4-bis-(diethylamino)-6-hydrazino-1,3,5-triazine
TMPP-AcPFP	S-pentafluorophenyl tris(2,4,6-trimethoxyphenyl)phosphonium acetate bromide
TMPP-PrG	4-hydrazino-4-oxobutyl)[tris(2,4,6-trimethoxyphenyl)phosphonium bromide
TCPH	2,4,6-trichlorophenylhydrazine
SALLE	salting-out assisted liquid–liquid extraction
DLLME	dispersive liquid–liquid microextraction
SIL-SPE-LC-DPIS-MS	stable isotope labeling-solid phase extraction-liquid chromatography-double precursor-ion scan mass spectrometry
DNLS-MS	double neutral loss scan mass spectrometry
HR-AM	high resolution accurate mass
MTS	mainstream tobacco smoke
EBC	exhaled breath condensate
ESI	electrospray ionization
APCI	atmospheric pressure chemical ionization
APPI	atmospheric pressure photoionization
UHPLC	ultra-high performance liquid chromatography
SWATH	sequential window acquisition of all theoretical fragment-ion spectra
QqTOF	quadrupole time-of-flight
UHR-FT MS	ultra-high resolution fourier transform mass spectrometry
LC-MS^n^	liquid chromatography tandem mass spectrometry
4-HNE	4-hydroxy-2-nonenal
4-HHE	4-hydroxy-2-hexenal
MDA	malondialdehyde
CID	collision-induced dissociation
HCD	high-energy C-trap dissociation
AIDA	alternate isotope-coded derivatization
